# Supplementary material for: High-resolution analysis of the treated coeliac disease microbiome reveals strain-level variation
Source: Gut Microbes. 2025 Apr 27;17(1):2489071. doi: 10.1080/19490976.2025.2489071 (PMC12036492; doi:10.1080/19490976.2025.2489071)
Supplement: Supplemental Material [file KGMI_A_2489071_SM0331.zip › SupplementaryFigures_v3.docx]

# Supplementary Figures


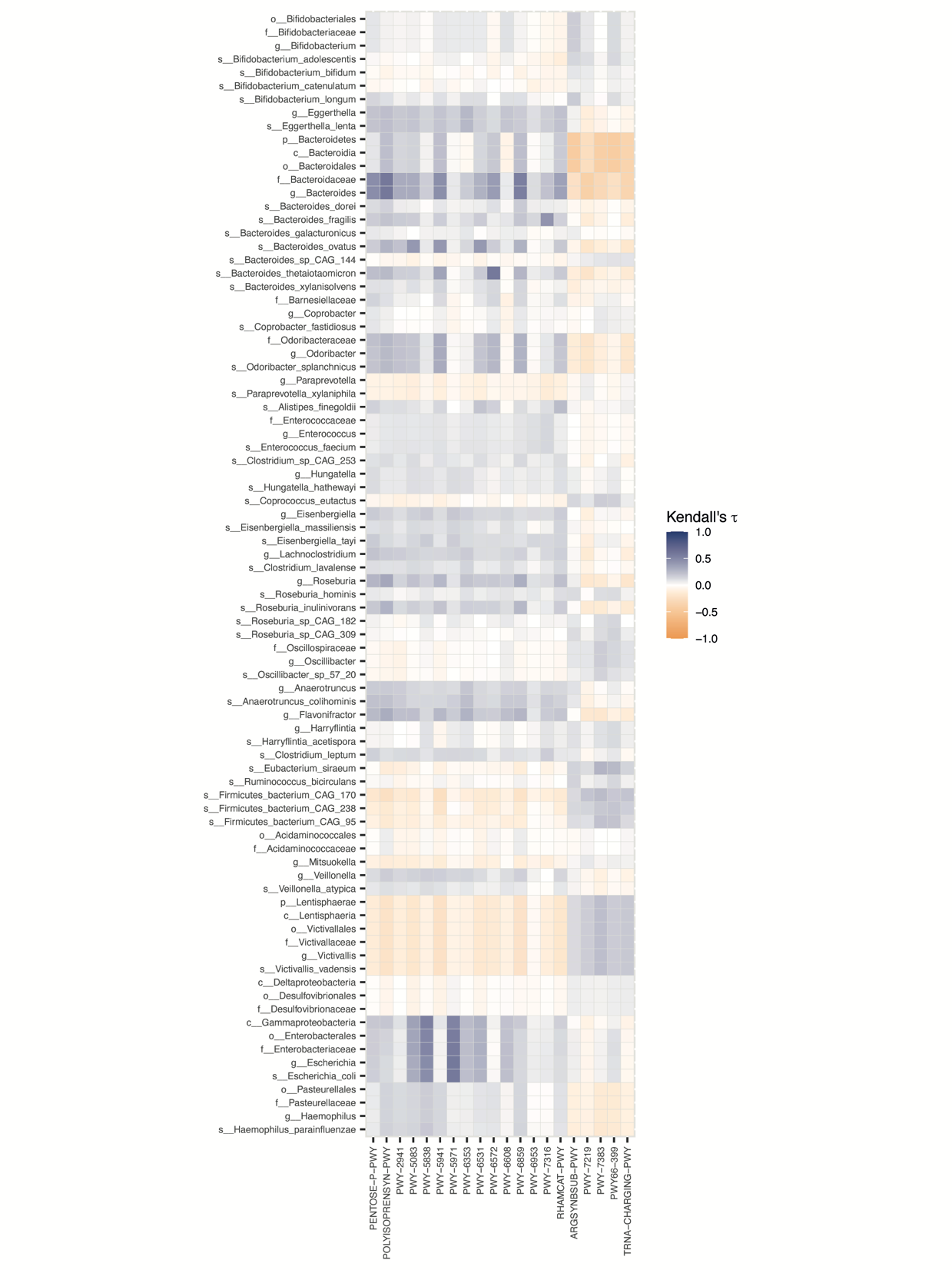


Supp. Figure S1: Correlation matrix between differentially abundant metabolic pathways (columns) and bacterial taxa (rows). The genus *Bacteroides* and higher-level taxa are included for visualization purposes.


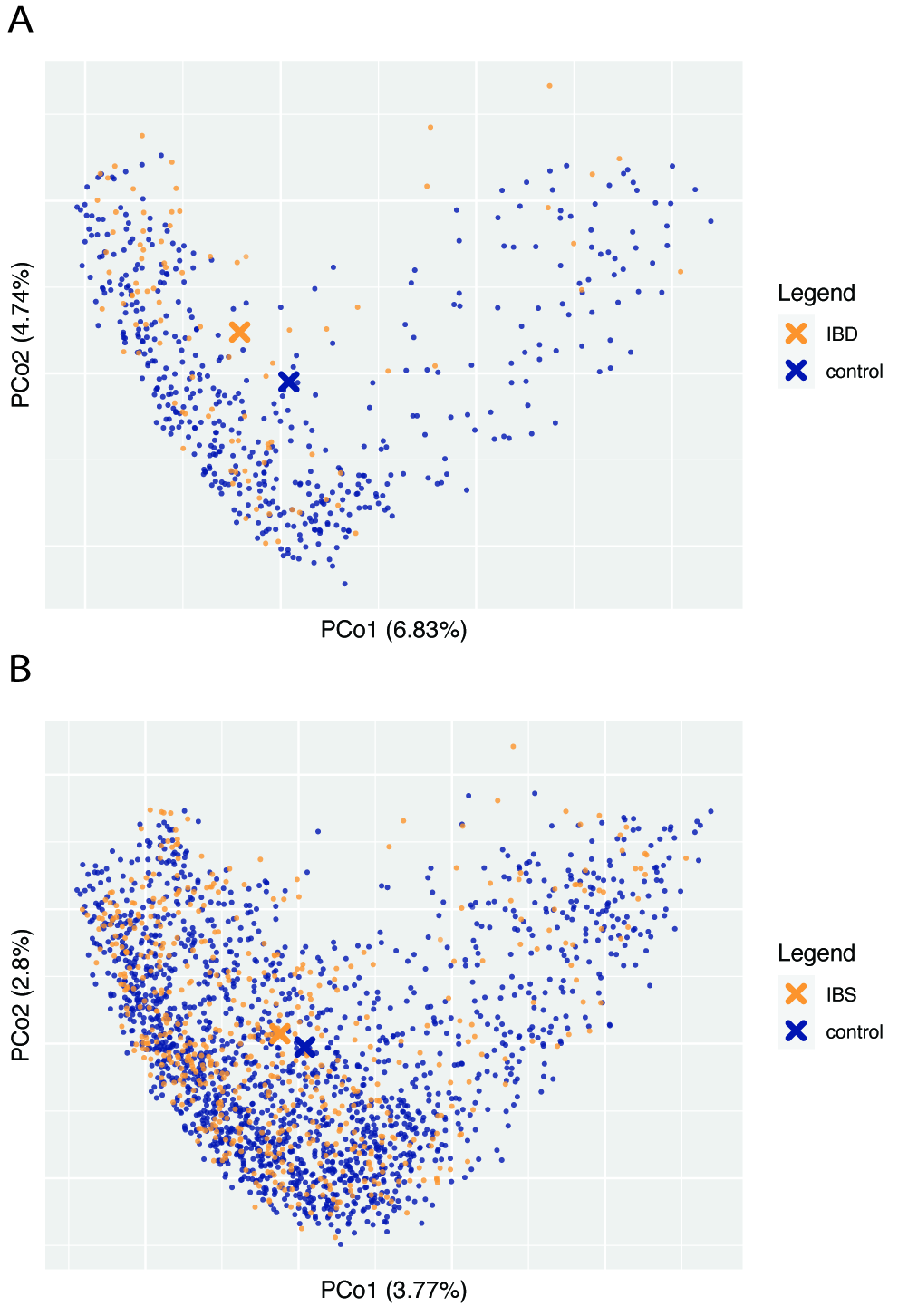


Supp. Figure S2: Beta diversity in IBD and IBS vs. controls, using Bray-Curtis dissimilarity as a distance metric. A. Principal coordinate plot of IBD samples and matched controls. Centroid locations are indicated with a cross. B. Principal coordinate plot of IBS samples and matched controls. Centroid locations are indicated with a cross.


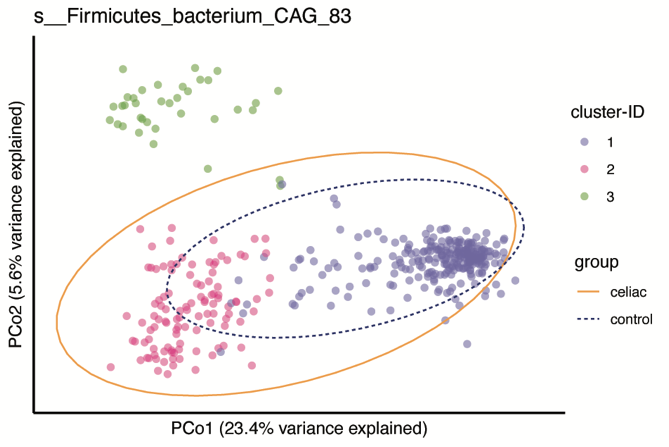


Supp. Figure S3: Non-metric multidimensional scaling plots of pairwise genetic distances between *Firmicutes bacterium CAG:83* strains. Points are colored according to their assigned cluster (PAM clustering). Ellipses include 95% of the samples in the indicated group.


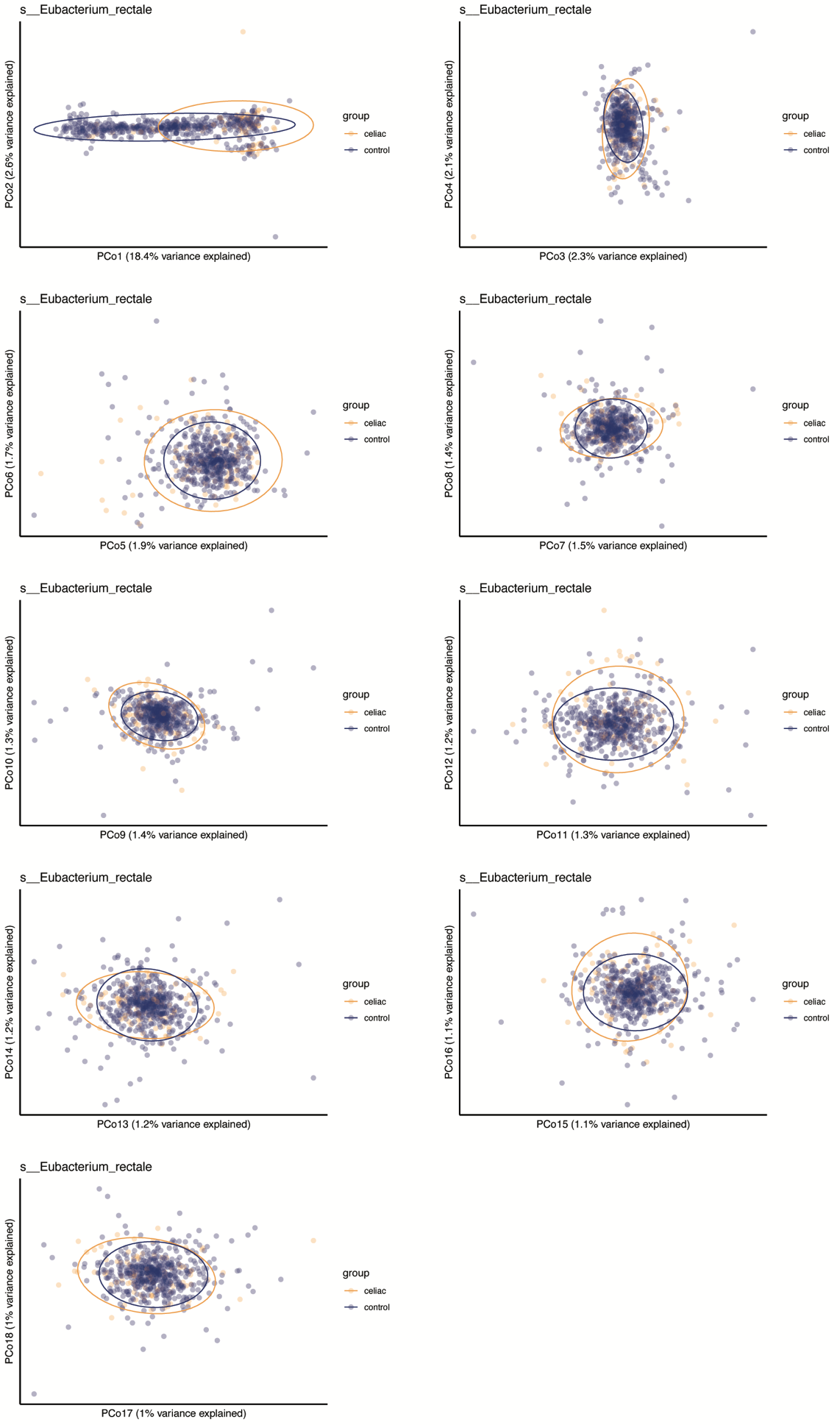


Supp. Figure S4: Non-metric multidimensional scaling plots of pairwise genetic distances between *E. rectale* strains, colored by disease status. All principal coordinates are shown that explain more than 1% of variation among samples. Ellipses include 95% of the samples in the indicated group.
